# HIV prevalence and behavioral risk factors in the Sudan People’s Liberation Army: Data from South Sudan

**DOI:** 10.1371/journal.pone.0187689

**Published:** 2017-11-21

**Authors:** Lauren P. Courtney, Norman Goco, John Woja, Tonya Farris, Chris Cummiskey, Emily Smith, Lia Makuach, Helen M. Chun

**Affiliations:** 1 BiostatEpi, RTI International, Washington, DC, United States of America; 2 BiostatEpi, RTI International, Research Triangle Park, North Carolina, United States of America; 3 HIV Secretariat, Sudan People’s Liberation Army, Juba, South Sudan; 4 Department of Defense HIV/AIDS Prevention Program, Naval Health Research Center, San Diego, California, United States of America; Public Library of Science, FRANCE

## Abstract

**Overview:**

After two decades of civil war, South Sudan has limited published data on HIV prevalence and behavioral determinants of HIV infection risk. A surge in HIV/AIDS prevalence is a real concern for this new country with limited access to medical or HIV preventive services, and low education and literacy levels. We present findings from the first bio-behavioral surveillance survey conducted within the Sudan People’s Liberation Army (SPLA).

**Methods:**

A cross-sectional survey of 1,149 randomly selected soldiers from thirteen SPLA bases was conducted in two phases: July to August 2010 and April to May 2012. Consenting participants received HIV rapid tests, pre- and post-test counseling, and a personal interview. Demographics, knowledge, attitudes, and behaviors, including sexual behavior, alcohol use, and mental health were assessed using computer-assisted interviews.

**Findings:**

The final sample included 1,063 survey participants (96.7% male). Education levels within the SPLA are low; only 16.4% attended school beyond the primary level. The overall HIV prevalence in the sample was 5.0% (95% confidence interval [CI]: 3.6–6.9). High-risk behaviors (e.g., multiple or concurrent sexual partners, heavy alcohol use, low condom use) were noted among SPLA members. High levels of HIV stigma were identified: 90.6% (n = 916) responded with one or more negative beliefs towards PLHIV, and 60.3% thought a healthy-looking person with HIV should not be allowed to remain in the SPLA.

**Conclusion:**

Results from this first evaluation of risk behaviors and HIV prevalence among the SPLA highlight high-risk behaviors that may contribute to the spread of HIV. Understanding potential comorbid conditions will be critical to designing strategies to reduce HIV risk. This survey represents the first steps in understanding the HIV epidemic within the SPLA context.

## Introduction

South Sudan has limited published data on HIV prevalence and behavioral determinants of risk for HIV infection. HIV prevalence in South Sudan is estimated at 3%, with approximately 150,000 to 200,000 South Sudanese living with HIV/AIDS [1]. Militaries may be at greater risk for HIV than the general population [2–7]. In this first bio-behavioral surveillance survey (BBSS) of the Sudan People’s Liberation Army (SPLA) conducted in 2010 and 2012, we sought to estimate the prevalence of HIV infection and understand related risk behaviors.

With the signing of the 2005 Comprehensive Peace Agreement between the Sudanese government and the SPLA, South Sudan ended two decades of civil war, which resulted in the displacement of nearly four million people, and near complete destruction of infrastructure, including health care [8–10]. Independence was celebrated on July 9, 2011. As refugees and foreign nationals return or enter the newly autonomous region, the potential arises for increased HIV prevalence [2,11].

Scarcity of HIV data in South Sudan has made it difficult to study HIV in specific demographics of the population [9,11]. Due to limited availability of HIV data, a conclusive picture of HIV prevalence in South Sudan cannot be established. However, in 2007 the Ministry of Health estimated HIV prevalence to be 3.1%, with 155,000 people living with HIV/AIDS (PLHIV) [10].

A surge in HIV/AIDS prevalence is a real concern as troops demobilize, displaced persons return home from years of dislocation, and commercial traffic from areas of higher HIV rates increases. Other potential drivers of the epidemic include a high level of illiteracy (97.5% among women aged 15 to 24 years) [10]; limited or no access to comprehensive HIV/AIDS prevention, care, and treatment services; and a low level of awareness and knowledge about HIV in the general population. According to the 2006 Southern Sudan Household Health Survey report, only 45.1% of women aged 15 to 49 years have ever heard of HIV, while only 9.8% knew the main three ways of preventing HIV transmission. In addition, only 35% of women knew that HIV can be transmitted through sexual intercourse [10]

The SPLA plays a central role in the government. It is the former armed wing of the Sudan People’s Liberation Movement, and constitutes the Armed Forces of the Republic of South Sudan. The mission of the SPLA is to defend the sovereignty of the country. Therefore, it is important that this population receive the necessary HIV/AIDS screening and education to prevent transmission, and early access to treatment.

The SPLA plays a significant role in efforts to reduce the impact of HIV in South Sudan not only within its military communities, but also in the broader community. The SPLA leadership recognized the threat of HIV and established the SPLA HIV/AIDS Secretariat in 2006, which coordinates, plans, implements, and monitors HIV/AIDS activities. The SPLA strategic plan for HIV/AIDS response aligns with the South Sudan HIV/AIDS Strategic Framework. Activities include the creation of an enabling environment, HIV prevention, care, treatment and impact mitigation, capacity building, and monitoring and evaluation.

This paper presents results from the 2010–2012 SPLA BBSS.

## Materials and methods

### Sampling/Participant selection

A cross-sectional survey of 1,149 randomly selected soldiers from eleven SPLA bases was conducted in two phases: 1. July to August 2010 and 2. April to May 2012. Thirteen possible sites were identified based on safety and accessibility: three sites in Central Equatoria (Bilfam, Mogiri, Joint Integrated Unit [JIU]), five in Western Equatoria (WE) (Yambio, Nzara, Tambura, Ezo, Maridi), two in Unity State (Renk, Duar), and one each in Western Bahr el Ghazal (Mapel), Northern Bahr el Ghazal (Wutnyiek), and Eastern Equatoria (Owiny Kibul). In phase 1, population proportionate to size sampling was used to select three out of the 6 non-Juba sites; all three Juba-based sites were included. In phase 2, five WE-based sites were purposely selected for inclusion. Random participant selection occurred during the daily base morning roll call, which was attended by troops of all ranks. Eligibility criteria included being aged ≥18 years, the ability to speak English or Juba Arabic, and membership in the SPLA or the JIU. After hearing the study description, eligible individuals who gave their free and informed written consent were assigned a unique subject identification number and an appointment time for their interview. Participants could consent to all or some of the activities, including interview, HIV rapid test, malaria and visceral leishmaniasis (VL) testing (2010), syphilis testing (2012), physical exam for circumcision in males, and dried blood spot card for HIV rapid test quality control (QC) and storage for future testing. Outside of this study, HIV testing was available to all soldiers through military base clinics, including those who did not wish to participate in this study.

#### Data collection methods

Computer-assisted personal interview (CAPI) and audio computer-assisted self-interview (ACASI) were used to collect behavioral data, and were administered at each base using portable touch screen netbooks. Trained interviewers used CAPI in either English or Juba Arabic to collect information for non-sensitive questions. ACASI eligibility criteria included basic literacy (English or Juba Arabic) and previous computer use. ACASI was used to ask sensitive questions to respondents eligible for this survey method (10.7% of participants, n = 119). Questions considered sensitive included topics related to alcohol use, sexual behavior, and sexual coercion. Ineligible ACASI respondents completed the entire interview using CAPI (89.3%, n = 993) in a private, secure setting. Since CAPI and ACASI are new methodologies for South Sudan, ACASI feasibility testing and questionnaire pretesting were conducted during a formative phase prior to field implementation.

#### Measures

The survey assessed demographics, military background, HIV knowledge and attitudes, HIV testing and treatment, sexual behavior, alcohol use, and mental health. Sections unique to each gender were included. Demographic characteristics included age, education, religion, marital history, and military background. HIV testing and treatment included questions regarding access to HIV testing, stigma, knowledge of one’s status or partner’s status, willingness to be tested, and access to and knowledge of antiretrovirals and other treatment.

Sexual behavior questions included condom access and use and partner type; recall was based on 3 months, 1 year, and 5 years, with an emphasis on behaviors in the last 3 months and the “last time had sexual intercourse.” Regular partners included a wife, girlfriend, or any person with whom a respondent had sex with three or more times or with whom the sexual relationship lasted >1 year. Casual partners included anyone with whom participants had sex only one or two times in the last 12 months. Multiple partners included respondents with three or more regular or casual partners in the past 12 months.

Alcohol use included the Rapid Alcohol Problems Screen 4-Quantity-Frequency (RAPS4-QF) [12]; a positive response signified screening positive for alcohol dependence. Mental health questions included a four-item Primary Care PTSD Screen (PC-PTSD) and a two-item depression screen, based on the Patient Health Questionnaire-2 (PHQ-2) [13].

Separate gender-specific sections explored attitudes, behaviors, and/or experiences regarding sexual coercion. The men’s section also included questions on circumcision.

#### HIV testing

Trained counselors used finger prick to conduct HIV rapid tests, following South Sudan’s national HIV testing guidelines at the time; parallel tests used Uni-Gold^™^ Recombigen^®^ HIV (Trinity Biotech, Bray Country, Ireland) and Determine^®^ HIV 1/2 (Inverness Medical Innovations, Petchabun, Japan) tests. Bioline^®^ HIV 1/2 3.0 (Standard Diagnostics, Inc., Korea) was used as a tiebreaker for discordant results.

Dried blood spot samples drawn by lancet from the finger were collected for HIV rapid test QC, malaria and VL testing in 2010, and syphilis testing in 2012, and storage for future testing.

#### Data processing and analysis

Data were weighted based on the probability of selecting the base and soldiers; weights were then adjusted for nonresponse. Basic demographics (military rank, education, marital status, years in military service, and age) were imputed using the Cox-Iannacchione weighted sequential hot deck method [14]. “Don’t Know” responses were recoded to incorrect answers for questions regarding participant’s knowledge of HIV; otherwise, “Don’t Know” responses were set to missing. All “Refuse” responses were set to missing. Weighted frequencies and means were calculated using appropriate weight adjustments, and variance estimates were calculated using the Taylor linear series based on the sample design [15]. All statistical calculations were conducted using SUDAAN (RTI International, Research Triangle Park, North Carolina, USA) and SAS software, version 9.2 (SAS Institute Inc., Cary, NC, USA).

#### Ethics and role of funding source

This research was approved by the Ethics Committees of the Government of South Sudan and RTI International (Protocol RTI IRB ID 12422). Naval Health Research Center (NHRC) provided technical input into the design and analysis of the study. NHRC approved submission of this manuscript for publication. Participants provided their written informed consent to participate in this study.

## Results

Of the 1,149 persons randomly selected to participate, 1,106 completed HIV testing (response rate 96.3%) and 1,063 completed the interview (92.5%). A total of 1,058 persons completed both the interview and HIV testing (92.1%).

### Demographic characteristics

Demographic characteristics and military background from male and female consenting participants are presented in Table 1. Approximately 55% of the sample were from three sites in Juba; the remaining participants were from eight sites outside of Juba: Mapel, Owinykibul, Duar, Yambio, Nzara, Tambura, Ezo, and Maridi.

**Table 1:** Demographics of SPLA survey participants.

Category (n = Total No. Respondents)	No. Respondents in Category	Percentage^a^
**Site (n = 1,063)**		
Juba: Bilfam	217	28.5
Juba: Mogiri	143	16.5
Juba: JIU	89	10.8
Mapel	103	11.0
Owinykibul	126	8.6
Duar	97	4.5
Yambio	63	4.1
Nzara	58	4.1
Tambura	64	3.8
Ezo	50	4.1
Maridi	53	3.9
**Gender (n = 1,063)**		
Male	1,020	96.7
Female	43	3.3
**Age (n = 1,063; mean: 34.82)**		
18–24	127	12.8
25–29	198	19.4
30–34	213	20.1
35–39	245	23.4
40–44	133	11.2
45–49	81	7.4
50+	66	5.7
**Local deployment >2 months (n = 1,043)**	439	42.5
**Military rank (n = 1,059)**		
Private	485	47.5
Non-commissioned officer	413	38.2
Officer (junior/senior/general)	161	14.3
**Length of time at base (n = 1,059)**		
<5 years	621	57.0
5+ years	438	43.0
**Highest education level (n = 1,060)**		
No school	331	30.4
Primary	545	53.2
Secondary/college/university	184	16.4
**Religious affiliation (n = 1,047)**		
Christian	973	93.6
Other (Muslim, none)	74	6.4
**Marital status (n = 1,058)**		
Never married	212	22.2
Currently married	801	73.5
Divorced	26	2.5
Widowed	19	1.8
If currently married, wives inherited (n = 762)	79	9.9
If currently married, multiple wives (n = 763)	306	40.1

^a^Weighted data.

The predominantly male sample had a mean age of 34.8 years, with the majority (62.9%) between the ages of 25 and 39. The average time in the military was 15.8 years. More than half (57%) had been stationed at their current base for less than 5 years, and nearly half had been on a local deployment for 2 months or more.

### HIV prevalence

HIV prevalence within this sample was 5.0% (95% confidence interval [CI]: 3.6–6.9) (Table 2). We conducted comparisons by HIV status, and found no significant differences due to the small sample size and prevalence rate.

**Table 2:** HIV prevalence among SPLA members.

HIV Status	Number	Percentage^a^	95% CI*
Positive	56	5.0	(3.6, 6.9)
Negative	1,044	94.5	(92.4, 96.0)
Indeterminate	1	0.1	(0.0, 0.6)
No test/refused	5	0.4	(0.1, 1.1)

*CI, confidence interval.

^a^Weighted data.

### HIV knowledge/stigma

Overall, SPLA respondents showed significant gaps in knowledge of HIV: 78.2% answered four or more of eight knowledge questions correctly and only 83 participants (7.1%) answered all questions correctly (shown in Table 3). High levels of HIV stigma were identified: 90.6% (n = 916) responded with one or more negative beliefs towards PLHIV, and 60.3% thought a healthy-looking person with HIV should not be allowed to remain in the SPLA. In addition, one quarter of respondents reported knowing a person who was denied military participation because he or she was suspected of being infected with HIV.

**Table 3:** Knowledge and stigma of HIV/AIDS among SPLA members.

Statement (n = total no. respondents)	No. Respondents Who Agreed With Statement	Percentage^a^	95% CI*
**Knowledge: Correct Responses to Questions About HIV**			
HIV cannot be transmitted from a mosquito bite (n = 1,047)	604	55.9	(51.6, 60.1)
A healthy-looking person can have HIV (n = 1,045)	591	56.3	(51.2, 61.3)
HIV risk can be reduced by having sex with only one faithful, uninfected partner (n = 1,046)	622	59.1	(55.5, 62.6)
HIV risk can be reduced by using a condom (n = 1,032)	599	57.9	(53.3, 62.5)
HIV can be transmitted from a mother to a child (n = 1,045)	636	60.5	(56.7, 64.1)
People can get HIV through circumcision, facial scarring (n = 1,046)	665	64.5	(60.6, 68.3)
HIV cannot be transmitted from sharing a meal with someone who is infected (n = 1,046)	708	66.7	(62.7, 70.5)
People cannot get HIV because of witchcraft or supernatural means (n = 1,046)	838	80.2	(77.3, 82.8)
**Stigma: Social Stigma Responses Toward Individuals With HIV**			
People with HIV/AIDS should feel ashamed (n = 1,040)	359	34	(30.5, 37.6)
Would want it a secret if family member infected with HIV/AIDS (n = 1,045)	478	44.8	(38.1, 51.6)
People who get infected with HIV/AIDS by having sex have gotten what they deserve since it is their own fault (n = 1,040)	533	51.6	(47.9, 55.3)
Teacher should not be allowed to continue teaching if infected with HIV/AIDS (n = 1,044)	532	51.5	(47.2, 55.8)
Would not buy fresh vegetables from a vendor who is infected with HIV/AIDS (n = 1,044)	544	53.1	(49.5, 56.6)

Note. “Don’t Know” responses for questions about HIV/AIDS knowledge were recoded to incorrect answers. “Don’t Know” responses for stigma were set to missing. All “Refuse” responses were set to set to missing.

*CI, confidence interval.

^a^Weighted data.

### HIV risk behaviors

Participants reported high-risk behaviors, including low condom use, probable alcohol dependence, multiple sexual partners, probable posttraumatic stress disorder (PTSD), and depression. More than half (54.4%) had never used or did not know if they had used a condom. Of those that ever used condoms (n = 457), 62.3% reported using a condom during their last sexual encounter. Probable alcohol dependence was high, with 37.4% testing positive on the RAPS4-QF scale. 23.1% of participants tested positive for symptoms of probable PTSD and 15.3% for symptoms of probable depression.

The prevalence of self-reported male circumcision was 50.9%. More participants reported the procedure having been conducted by a medical provider versus a traditional healer (54.7% and 42.8%, respectively). Of those not circumcised (n = 443), 42.9% reported being willing to have the procedure if offered.

Men’s behaviors and attitudes towards sexual coercion included 20.6% believing women are raped because of something they said or did and 36.2% believing sex is human nature and that women should give it to men. 14% of men reported threatening to use force to get a woman to have sex, while 2% of men reported having ever forced a woman to have sex (Table 4). Note that this question was added for phase 2 of the survey implementation, and thus, the smaller sample size.

**Table 4:** HIV risk factors among SPLA participants.

Category (n = Total No. of Respondents)	No. of Respondents in Category^a^	Percentage^b^	95% CI^b^
Men never used a condom or replied “Don’t Know” (n = 989^c^)	540	54.5	(47.2, 61.6)
Men who have used a condom but did not use condom last time had sex (n = 457)	176	37.7	(32.5, 43.1)
Alcohol use in past 3 months (n = 1,053)	437	42.8	(36.3, 49.6)
Alcohol dependence based on RAPS4-QF (n = 1,041)	375	37.4	(30.9, 44.4)
Multiple partners in past 12 months (n = 867)	198	22.7	(19.6, 26.2)
PTSD, based on PC-PTSD (n = 1,035)	232	23.1	(20.3, 26.3)
Depression, based on PHQ-2 (n = 999)	145	15.3	(12.8, 18.3)
Sexual coercion: Ever threatened to use force to get woman to have sex (n = 726)	106	14.13	(9.6, 20.4)
Sexual coercion: Used force to get woman to have sex in the past year (n = 288)	6	2.0	(0.9, 4.8)
Men uncircumcised (n = 997)	470	49.1	(44.8, 53.5)
HIV knowledge <100% of 8 questions correct (n = 1,063)	980	93.1	(90.9, 94.7)
HIV stigma; positive response on at least 1 of 3 questions (n = 1,015)	916	90.6	(87.8, 92.7)

CI, confidence interval; PC-PTSD, Primary Care PTSD Screen; PHQ-2, Patient Health Questionnaire 2-question screen; PTSD, posttraumatic stress disorder; RAPS4-QF, Rapid Alcohol Problems Screen 4-Quantity-Frequency.

^a^n indicates the total number responding “yes” to the question.

^b^Weighted data.

^c^n indicates the total number responding to each question (varying because of questionnaire skip logic).

Examining alcohol as a risk factor, of those who drank in the last 3 months (42.8% “drinkers”), 61.6% engaged in high-risk behaviors, and 86.0% scored positive for probable alcohol dependence. Drinking prevented use or correct use of condoms in the last 3 months for 29.5% of participants reporting alcohol use. In the last 3 months, 28.3% of drinkers reported blacking out, and 30.5% had unintended sex after drinking.

## Discussion

### Major findings

This is the first BBSS conducted in the SPLA, and results suggest the current HIV prevalence among the SPLA is higher than the limited national estimates for HIV prevalence and lower than neighboring countries, including Kenya and Uganda, and higher than Ethiopia and DRC. In 2012 HIV prevalence from antenatal clinics and voluntary counseling and testing (VCT) clinics in Western Equatoria State (WES) was found to be higher than in other states (10.7% among antenatal clinics with 95% CI of 8.0–14.2%; 13.1% among VCT clinics with 95% CI of 10.0–17.0) [16]. Our HIV prevalence data for this region are slightly lower, ranging from 3.8 to 4.1 for the 5 SPLA bases tested in WES. Risk factors of concern for HIV include a low level of HIV knowledge and a high level of stigma. Other risk factors of note were a high percentage of multiple partners, low use of condoms, and high rates of probable alcohol dependence. Additionally, in this post-civil-war population, high rates of probable depression and PTSD were found, both of which have been linked to high-risk sexual behaviors and the potential risk for increased rates of HIV and other sexually transmitted infections [17–21].

Rapid spread of HIV infection in South Sudan may result from the complex interaction of current social, economic, geopolitical, cultural, and infrastructure-related factors existing prior to and following independence. The SPLA will continue to remain a highly mobile population for the near future, and these factors may serve as efficient bridges of HIV transmission between and within their communities.

#### HIV knowledge/stigma

HIV education within the SPLA, including knowledge of HIV transmissibility, could be improved. SPLA survey participants scored low when asked basic questions about HIV transmissibility, with very high rates of misconception (99% had at least one misconception). Rates were comparable to similar studies in African militaries, as in a 2002 study of the Ethiopian military [22]. As a result of the post-conflict setting in South Sudan, education and literacy in this population are extremely low: 30.4% never attended school, and overall, only 27% of South Sudan is literate [23]. Low literacy presents significant challenges to HIV prevention and education in this population.

High levels of stigma toward PLHIV persists in the SPLA: 60.3% reported that healthy-looking HIV-positive soldiers should not be allowed to stay in the military, which is comparable to data from one Ethiopian military study conducted almost a decade earlier (63%) [21]. Fear of stigma is associated with lower utilization and acceptance of services for HIV testing, care, and treatment [24]. High rates of stigma may lead to barriers for implementation of any SPLA HIV policies aimed at creating an environment free of stigma and discrimination.

#### Risky sexual behaviors

SPLA members participate in many high-risk sexual practices that threaten the spread of HIV. Condom use is relatively low, with only 45.6% reporting ever using a condom, of which, only 62.6% during their last sexual encounter. In rural regions (outside of Juba), condom use is considerably lower (as low as 15% in one rural base). Condom promotion and access and education on correct use are still greatly needed.

As a cultural practice, having multiple wives is a common occurrence in South Sudan. Furthermore, although not openly discussed, having multiple partners is also quite common. With more disposable income, along with high mobility, men in the SPLA may have more opportunities to have sexual relations with partners and commercial sex workers than the general population. These practices place SPLA members at increased risk for HIV infection.

#### PTSD and depression in the SPLA

Research conducted after military conflicts has shown that deployment and exposure to combat result in increased risk for PTSD, depression, and substance abuse. We found notable levels of probable PTSD and depression within the SPLA, which were slightly higher than published results from the United States and other militaries with recent deployment or conflict experience. One study of American soldiers and marines who recently returned from Iraq showed that 17% screened positive for PTSD, generalized anxiety, or depression [25]. Another study showed that 19.1% of U.S. service members returning from Iraq, and 11.3% of those returning from Afghanistan, reported mental health problems [26]. In South Sudan, one report of civilians showed a 48% prevalence of PTSD [27]. Although over 5 years have passed since the 2005 Comprehensive Peace Agreement was signed in Sudan, PTSD continues to significantly affect this population. PTSD has been associated with high-risk sexual behaviors [19–21]. Understanding potential comorbid conditions will be critical for designing strategies to reduce HIV risk.

#### Alcohol use

In this study, probable alcohol dependence among SPLA soldiers was high. In a sample of Dominican Republic military personnel, alcohol use was associated with inconsistent condom use with casual partners, greater number of lifetime and multiple or concurrent sex partners, and transactional and coercive sex [19]. In addition to addressing the well-being of its soldiers, given the intersection between alcohol and HIV risk, interventions to reduce heavy alcohol use among SPLA soldiers will be critical in the HIV response.

### Limitations

Significant differences between groups by HIV status were not observed, primarily due to the limited sample size. Survey results represent the fourteen bases included in the original sampling plan. Bases in conflict settings were excluded because of reduced security. Data collection in Western Equatoria took place at a later time, resulting in a two-year gap in data collection. The number of women in the study was small (n = 43), limiting the potential for analyses by gender. No data were collected on soldiers who refused to participate (7.9%), and could represent a larger proportion of HIV positives. Finally, participants often declined to include their date of birth or provided inaccurate dates.

### Conclusions

These data from the first SPLA BBSS represent key first steps in understanding the HIV epidemic in the specific context of the SPLA. This will help inform current efforts for HIV prevention and policy, as well as identify areas for future evaluation. Additional research within the SPLA is needed to gain a better understanding of HIV prevalence by region, gender, and other key demographics, and the role of occupational and mental health factors, such as depression, PTSD, and alcohol use in sexual risk behaviors and the risk of HIV and other STI acquisition. The SPLA have used these results to improve prevention programming. For example, following the identification of potential high risk of alcohol abuse, they prioritized the development of an alcohol-focused risk reduction program to address the potential increased risk from alcohol use. The SPLA recognizes the key role it plays in the response to the HIV epidemic in South Sudan and continues to take steps toward addressing the challenges posed through its commitment to further examination of HIV and risk behaviors, operations research related to alcohol, and evaluation of the structural requirements to provide quality services in HIV prevention, care, and treatment.
